# Analysis of the Composition and Phylogenetic Relationships of the *Acanthosaura coronata* Complex Including Molecular Identification of Historical Specimens

**DOI:** 10.3390/ani16081261

**Published:** 2026-04-20

**Authors:** Natalia B. Ananjeva, Maryia I. Matsiushova, Anton O. Svinin, Olga S. Bezman-Moseyko, Luan Nguyen Thanh, Nikolai L. Orlov

**Affiliations:** 1 Department of Herpetology, Zoological Institute, 199034 Saint-Petersburg, Russia; m.matsiushova@gmail.com (M.I.M.); bezman-moseyko@mail.ru (O.S.B.-M.); orlov52@gmail.com (N.L.O.); 2 Institute of Cytology, Russian Academy of Sciences, 194064 Saint-Petersburg, Russia; ranaesc@gmail.com; 3 Australian Museum Research Institute, Australian Museum, 1 William St, Sydney, NSW 2010, Australia; luan.nguyen@australian.museum; 4 Centre for Ecosystem Science, School of Biological, Earth and Environmental Sciences, University of New South Wales, Sydney, NSW 2052, Australia

**Keywords:** Agamidae, *Acanthosaura*, molecular analysis, systematic, phylogeny, mitochondrial DNA

## Abstract

The genus *Acanthosaura* is characterized by a high level of cryptic species diversity and is subdivided into several species complexes. The phylogenetic relationships within the *A. coronata* complex remain unresolved due to the presence of cryptic lineages and limited molecular data for several species. In this study, these relationships are clarified using a molecular genetic analysis that integrates newly collected field samples and historical museum specimens with previously uncertain identification. Three mitochondrial genes (*cyt b*, *COI*, and *ND2*) from samples, including fresh collections of *A. murphyi* from Phu Yen Province (Vietnam) and museum specimens from Vietnam and Myanmar, were analyzed. In addition, morphological characters of the examined specimens with diagnostic traits of known species were compared. Phylogenetic analyses confirmed the distinct species status of *A. murphyi* and enabled the taxonomic reassignment of previously undetermined museum specimens to this species. Specimens from Vietnam and Myanmar formed a single, well-supported clade, suggesting a broader distribution for *A. murphyi* than previously recognized. It is demonstrated for the first time that *A. murphyi* belongs to the *A. coronata* complex, together with *A. coronata* and *A. cuongi*, a result consistently supported by both genetic distances and phylogenetic tree topology.

## Simple Summary

The genus *Acanthosaura*, a group of rainforest agamid lizards from Southeast Asia, contains many morphologically similar but genetically distinct species, making their classification difficult. In this study, we used molecular analysis to clarify the relationships within one problematic group, the *A. coronata* complex. We analyzed freshly collected lizards in Vietnam and old museum specimens collected decades ago in Vietnam and Myanmar. By comparing three mitochondrial genes, we were able to confirm that a species called *A. murphyi* is genetically distinct and belongs to the same group as *A. coronata* and *A. cuongi*. We also solved a long-standing puzzle by identifying old, unnamed museum specimens as *A. murphyi*. This finding shows that *A. murphyi* lives in a much larger area than previously thought, ranging from central Vietnam to Myanmar. Our work highlights how combining a new field study with DNA analysis of museum collections is essential for discovering and classifying hidden biodiversity.

## 1. Introduction

The family Agamidae currently comprises 610 species belonging to 64 genera and 6 subfamilies [1]. The majority of agamid lizard species (293 species, i.e., 48%) belong to the subfamily Draconinae Fitzinger, 1843, which includes 33 recent genera of agamas. Conceptions of the phylogenetic and taxonomic diversity of dragonin agamids are constantly evolving. For instance, the genus *Japalura* sensu lato has long been recognized as paraphyletic, and only a new revision incorporating multilocus phylogeny and morphological analysis has provided the first phylogenetic inference of relationships among *Japalura* s. l. species [2]. The authors concluded that four major clades should be distinguished. There are 9 species of *Japalura* Gray, 1853 sensu stricto, and one of the most species-rich genera, *Diploderma* Hallowell, 1861, with 50 recognized species. They also described the genus *Cristidorsa* Wang, Che, Lin, Deepak, Datta-Roy, Jiang, Jin, Chen et Silver, 2018 with two species. Finally, *J. bapoensis* was referred to the genus *Pseudocalotes* Fitzinger, 1843. Genus *Pseudocalotes* (23 species), is polyphyletic and is represented by at least two distinct genera [3,4]. The species *Pseudocalotes austeniana* (Annandale, 1908), previously considered within this genus, was reassigned to *Japalura* [4]. A recent revision of the genus *Phoxophrys* Hubrecht, 1881 revealed to be paraphyletic and revalidated *Pelturagonia* Mocquard, 1890 for all Bornean species of this genus [5].

The genera of the subfamily Draconinae are continually being enriched with new species of agamas [4,6,7,8,9,10,11,12], including those considered extinct for centuries [13]. This is largely due to the active application of molecular methods, which allow for the identification of cryptic diversity within taxa. An ever-increasing number of species represents the genus *Acanthosaura* Gray, 1831. Some of them are cryptic species, for which it is often difficult to identify clear morphological differences [6,7], which has historically led to a number of problems in their system that can only be resolved using molecular methods.

Currently, the genus *Acanthosaura* Gray, 1831 comprises 22 recognized species [1], and is typically assigned to several species groups (complexes), in addition to some species with uncertain positions for which molecular data are lacking [7]. Thus, 17 of 22 species were described in the 21st century, a period marked by expanded opportunities for new materials and the implementation of advanced molecular methods.

However, some problems also persist regarding a number of specimens examined using molecular methods, preventing a clear resolution of phylogenetic relationships within the groups and their composition. In 2004, two *Acanthosaura* specimens originating from an unknown locality in Myanmar were recorded [14]. Subsequent molecular analysis (cyt *b* gene) revealed their distinct phylogenetic position within an undefined group of *Acanthosaura* (the cysteine lineage) [14]. In 2020, new *cyt b* data for *A. coronata* allowed its placement within the cysteine lineage, together with the two Myanmar specimens and a misidentified *A. crucigera* isolate ROM37083 from Dong Nai, Cat Tien National Park, Vietnam [6]. In 2018, *A. murphyi* was described, showing genetic similarity (based on the *COI* marker) to the *Acanthosaura* specimen BGM01, which was preliminarily identified as *A. capra* (MK239022) [15]. In the phylogenetic tree based on *COI*, *A. murphyi* occupies a similar sister position to *A. coronata* as the two Myanmar specimens in the *cyt b* tree [7]. Thus, resolving the taxonomic status and relationships of these lineages requires the examination of *A. murphyi* specimens using at least two genetic markers.

Historically, the grouping of *A. capra*, *A. nataliae*, and *A. murphyi* into a single complex was justified by their external morphological similarities [6,14,16]. Namely, all three species of the *A. capra* complex are large-sized agamids possessing only a single pair of postorbital spines, in contrast to the large species *A. armata* and other congeners, which have two pairs of spines (postorbital and nuchal or occipital). In this context, the isolated position of *A. murphyi* is particularly intriguing. *Acanthosaura murphyi* is a narrow-range endemic species currently known from areas east of the distribution range of *A. capra* in Vietnam, specifically in Hon Ba Nature Reserve, Khanh Hoa Province, and Ca Forest, bordering Phu Yen and Khanh Khoa provinces. This species has been recorded in more recent collections in Song Hinh Commune, Phu Yen Province, Vietnam.

This study aims to clarify the relationships within the *A. coronata* complex by conducting a more complete molecular genetic analysis of three genes (*cyt b*, *COI* and *ND2*) using newly obtained fresh material of *A. murphyi*, as well as museum specimens with disputed or uncertain identification.

## 2. Materials and Methods

### 2.1. Sample Collection

**Figure 1:**
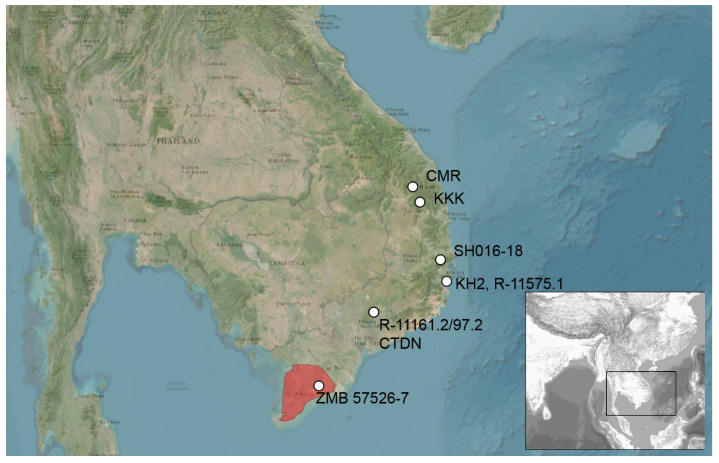
Collection localities of the material used in this study. The red area illustrates the territory south of the Mekong Delta, a more detailed locality of the specimens with ZMB vouchers is unknown.

The following specimens were used in the molecular genetic analysis (Figure 1): three specimens of *A. murphyi* ILS H 2922–2924 (vouchers SH-016-018) newly collected from Song Hinh Commune, Phu Yen Province, Vietnam (2019); two historical specimens identified as *A. capra* ZMB 57526-27, Vietnam (collected by U. Manthey in 1997); two specimens identified as *A*. sp. A94, A95 (HLMD-RA2969-26970), from the pet trade in Myanmar, sequenced earlier [14]; and two samples of *A. grismeri* ZMMU R-11575.1 and R-11539 (Supplementary Table S1). For several *A. cuongi* (vouchers KKK53, KKK55, KKK108-109, CMR88-90) and *A. coronata* (vouchers CTDN2, CTDN65, CTDN67, CTDN97, CTDN201, CTDN209) from our previous study [7], for which *cyt b* and *COI* sequences were available, *ND2* sequences were also obtained. Other sequences of *Acanthosaura*, including data from our research [7], were downloaded from GenBank NCBI. The morphological characters of historical museum specimens were also examined for comparison with the diagnostic characters of *A. murphyi*.

### 2.2. Morphological Analysis

A set of characters was selected to ensure the maximum comparability of published data for all currently recognized species of the genus *Acanthosaura*. Comparative morphological data were taken from original descriptions and subsequent studies [6,11,14,15,16] including comprehensive summarized tables with data on all the species of the genus *Acanthosaura* [17]. All measurements were taken using dial calipers on the right side of the body (in millimeters [mm]) to the nearest 0.1 mm; morphometric studies were carried out in accordance with [15]): SVL—snout-vent length, measured from the tip of the snout to the tip of the vent; TAL—tail length, measured from the posterior margin of the vent to the tip of the tail; TBW—tail base width, maximum width at tail base; TAL/SVL—ratio of tail length to snout-vent length; HL—head length, measured from posterior edge of the rectis of the jaw to the tip of the snout; HW—maximum head width, the width at the level of the tympanum; HD—maximum head height, measured across the parietal region; SL—snout length, measured from the anterior edge of the orbit to the tip of the snout; ORBIT—orbit diameter, measured from the posterior to the anterior edge of the orbit; EYE—eye diameter, measured from the posterior to the anterior edge of the eye; TD—tympanum diameter, measured horizontally from the anterior to the posterior border of the tympanum; PS—postorbital spine length, measured from the base to the tip; PS/HL—ratio of postorbital spine length to head length; NSL—maximum length of the largest spine in the nuchal crest measured from the base to the tip; NSL/HL—ratio of nuchal spine length to head length; DS—maximum length of the largest spine in the dorsal crest, measured from the base to the tip; WNC—maximum width of the spines in the nuchal crest, measured at the base; WDC-maximum width of the spines in the dorsal crest, measured at the base; DIAS—length of the diastema, measured from the posterior end of the nuchal crest to the anterior end of the dorsal crest; DIAS/HL—ratio of diastema length to head length; FOREL—forelimb length, measured from axilla to the proximal edge of the palmar region; HINDL—hindlimb length, measured from groin to the proximal edge of the plantar region; RW—rostral width; RH—rostral height; MW—mental width; MH—mental height. Meristic traits (pholidosis): SN—number of spines on the nuchal crest; DIASN—number of scales in the vertebral crest scale diastema, counted from the posterior end of the nuchal crest to the anterior end of the dorsal crest; CS—number of canthus rostralis—supraciliary scales, counted from the nasal scale to the posterior end of the ridge at the posterior margin of the orbit; SUPRAL—number of supralabials; INFRAL—number of infralabials; VENT—number of ventral scales counted at the midline from the anterior edge of the shoulders to the edge of the vent; FI—number of subdigital lamellae on the fourth finger; RS—number of scales bordering the rostral scale; NS—number of scales between the nasals; NCS—number of scales between the fifth canthals; NSCSL—number of scales from the fifth canthal to the fifth supralabial; NR—number of scales between the nasal and the rostral. We also measured the frequency of YAS, presence (1) or absence (0) of a Y-shaped arrangement of enlarged scales on the snout, and GP, the size of the gular pouch, scored as absent (1), small (2), medium (3), or large (4).

### 2.3. Molecular Analysis

Tissue samples consisted of muscle and skin fragments, which were collected from the examined specimens using sterile instruments and preserved in 70% ethanol. Prior to DNA extraction, the samples were air-dried to remove ethanol and homogenized in lysis buffer. DNA was extracted using the Biolabmix DU-250 kit (Biolabmix, Novosibirsk, Russia). DNA concentration was measured using a Micro Spectrophotometer Nano-500 (Allsheng Instruments, Hangzhou, China), and samples with a concentration of at least 10 ng/µL were selected for further analysis. Three mitochondrial gene fragments were used in the study: the cytochrome *b* gene (*cyt b*), the cytochrome *c* oxidase subunit I gene (*COI*), and the NADH dehydrogenase subunit 2 gene (*ND2*). The primers used are provided in Table 1.

The PCR reaction mixture (25 µL) contained 50–100 ng of DNA, 1 µM of each primer, 0.2 mM dNTPs, 1.5 mM MgCl_2_, 2.5 µL of 10× PCR buffer (10 mM Tris-HCl, pH 8.3, 50 mM KCl), and two units of Taq polymerase (Master Mix, AlcorBio, Saint-Petersburg, Russia). The PCR protocol for amplifying the cytochrome *c* oxidase subunit I fragment (*COI*) included an initial denaturation step at 95 °C for 15 min, followed by 34 cycles of denaturation at 95 °C for 30 s, annealing at 56 °C for 30 s, extension at 72 °C for 1 min, and a final extension at 72 °C for 5 min [18]. The protocol for *cyt b* included an initial denaturation at 95 °C for 15 min, followed by 35 cycles of denaturation at 95 °C for 30 s, annealing at 52 °C for 30 s, extension at 72 °C for 30 s, and a final extension at 72 °C for 5 min [19]. The protocol for *ND2* included step at 95 °C for 15 min, followed by 35 cycles of denaturation at 95 °C for 30 s, annealing at 52 °C for 30 s, extension at 72 °C for 30 s, and a final extension at 72 °C for 5 min [20,21]. The PCR products were purified using the MagPure Gel Pure DNA Kit (Magen Biotech Co., Guangzhou, Guangdong, China). Sequencing was performed on an ABI 3500 automated sequencer (Applied Biosystems, Life Technologies, Waltham, MA, USA) using the BigDye^®^ Terminator v. 3.1 kit (Applied Biosystems) with the same primers used for amplification (Evrogen, Moscow, Russia). Sequences were deposited in GenBank NCBI under the accession numbers PZ027938-46 and PZ056157-77.

### 2.4. Phylogenetic Analysis

The obtained sequences were manually aligned using Chromas version 2.5.1 (Technelysium Pty Ltd., South Brisbane, QLD, Australia). Individual gene alignments were then generated with MAFFT version 7.526 using the FFT-NS-2 strategy [22,23]. For Draconinae subfamily phylogeny analysis, the three genes (*ND2*, *COI*, *cyt b*) were concatenated according to their occurrence in the *Acanthosaura lepidogaster* mitogenome (KR092427). To reconstruct the phylogeny of the subfamily Draconinae, we used mitochondrial genome data. From these data, only the genes examined in our study were selected for alignment, concatenation, and phylogenetic inference. Following alignment, a maximum likelihood phylogeny was inferred with IQ-TREE 3.0.1 [24]. Bayesian trees were constructed using the MrBayes v. 3.2 software [25]. The best-fit substitution model was selected automatically by ModelFinder [26] according to the Bayesian Information Criterion (BIC). Branch support was assessed with 1000 ultrafast bootstrap (UFBoot) replicates and the Shimodaira-Hasegawa-like approximate likelihood ratio test (SH-aLRT). Finally, uncorrected *p*-distances were calculated with 1000 replicates in MEGA X v. 10.2.2 [27] to estimate pairwise genetic divergence for sequences grouped by species and genus. Standard population genetic parameters for each gene were calculated in DnaSP version 6.12.03 [28].

## 3. Results

### 3.1. Phylogenetic Relationships

**Figure 2:**
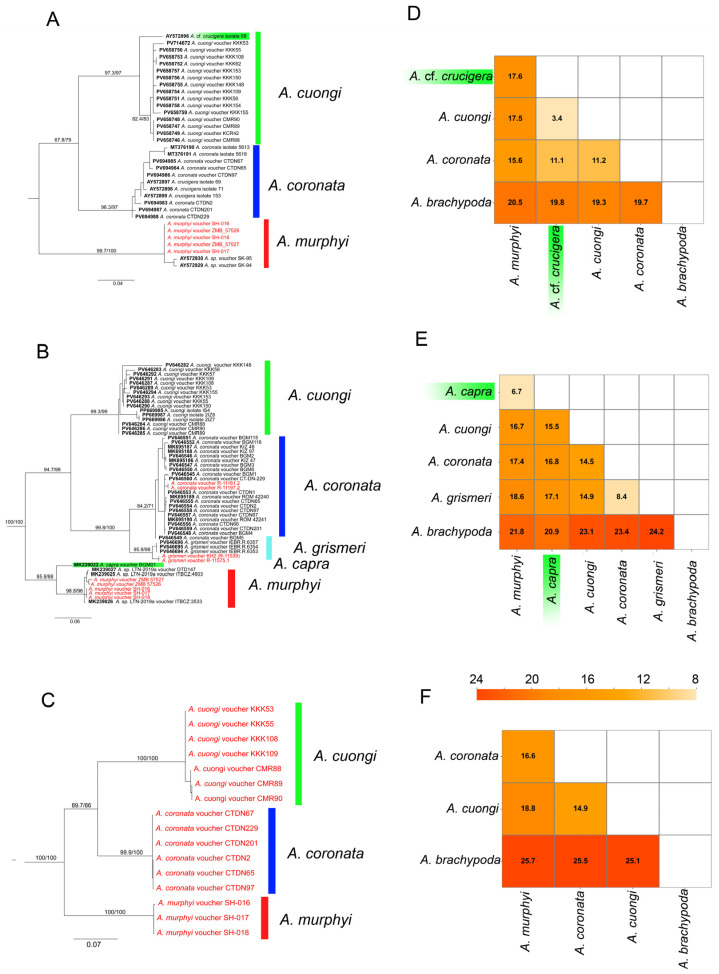
Phylogenetic reconstruction of the *Acanthosaura coronata* species group based on three mitochondrial gene fragments. The cytochrome *b* tree (**A**) was inferred under the TPM3u+F+G4 model, with branch support assessed using UFBoot/SH-aLRT replicates (1000 replicates each). The *COI* tree (**B**) was obtained from an alignment of 545 bp (188 parsimony-informative sites) under the best-fit model TIM3+F+G4; support values are likewise based on 1000 UFBoot/SH-aLRT replicates. The *ND2* tree (**C**) was reconstructed under the TN+F+G4 model from an alignment of 1032 bp (358 parsimony-informative sites). The heat maps (**D**–**F**) represent uncorrected *p*-distances (for *cyt b*, *COI*, *ND2*, respectively) between mitochondrial haplotypes, illustrating levels of genetic divergence within and between putative species of the *A. coronata* complex. Our *A. brachypoda* sequences used as the outgoups. Our sequences are highlighted in red.

Phylogenetic analyses of the three mitochondrial genes recovered identical topologies with robust support for both nodes and branches (Figure 2A–C). The acanthosaurs examined in this study cluster into three well-supported clades based on the cyt *b* and *ND2* genes, corresponding to the three species *A. coronata*, *A. cuongi*, and *A. murphyi*. The phylogeny based on *COI*, which includes several additional specimens not studied for the other two markers, reveals the existence of five clades. Three of these are identical to those in the *cyt b* and *ND2* phylogenies, while the other two represents the branches corresponding to the recently described species *A. grismeri* [29] and one sequence of *A. capra* [15] (Figure 2B).

The *A. murphyi* sequences we obtained (vouchers SH-016-18 and ZMB 57526-27) were placed within the *A. murphyi* clade in the *COI* phylogeny. In the *cyt b* phylogeny, they also formed a single clade with samples SK94-95 from Myanmar, which undoubtedly assigns them to *A. murphyi*. The *ND2* phylogeny, represented exclusively by our samples, confirmed the separation of the *A. murphyi* clade. Two specimens from the ZMMU collections (vouchers R-11539 and ZMMU R-11575.1) belong to *A. grismeri*.

**Figure 3:**
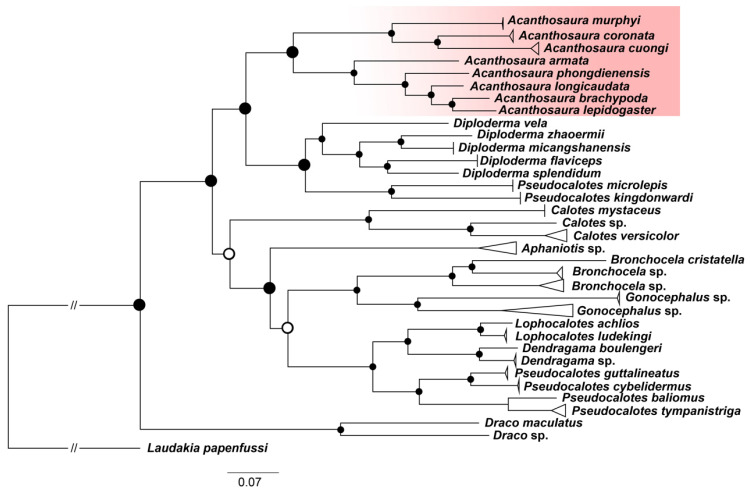
Phylogenetic reconstruction of the subfamily Draconinae based on three concatenated mitochondrial gene fragments (*ND2*, *COI*, *cyt b*). The mitochondrial tree was inferred from an alignment of 1935 bp (1214 parsimony-informative sites) under the best-fit model GTR+F+I+G4, with branch support assessed using UFBoot/SH-aLRT replicates (1000 replicates each). The three genes (*ND2*, *COI*, *cyt b*) were concatenated according to their occurrence in the *Acanthosaura lepidogaster* mitogenome (KR092427). Node support values below 70% are indicated by white circles with black outlines, while values above 90% are shown in black. Acanthosaura sequences are highlighted in red.

The calculated interspecific genetic distances based on *COI* revealed substantial *p*-distances between *A. murphyi*, *A. coronata*, and *A. cuongi*, ranging from 14.4% to 17.4% (Figure 2D). In contrast, the uncorrected *p*-distance between *A. murphyi* and *A. capra* was notably lower at 6.7%, while the divergence between *A. coronata* and *A. grismeri* was 8.4% (Figure 2D). The phylogeny based on the three concatenated mitochondrial genes recovered a consistent branching topology within the investigated species group (Figure 3).

### 3.2. Morphology of Species from Acanthosaura coronata Complex

As previously mentioned, our analysis of three mitochondrial genes (*cyt b*, *COI* and *ND2*) from various samples, including fresh collections of *A. murphyi* from Phu Yen Province in Vietnam and museum specimens from Vietnam and Myanmar, revealed that *murphyi* belongs to the *A. coronata* species complex for the first time. We also demonstrated that a significant discrepancy has been identified in the interpretation of the species complex composition, arising from contrasting morphological and molecular analyses. The grouping of *A. capra, A. nataliae* and *A. murphyi* into a single complex was justified by their external morphological similarities. Specifically, all three species of the *A. capra* complex are large agamids with only one pair of postorbital spines. This is in contrast to the larger species *A. armata* and other congeners, which have two pairs of spines (postorbital and nuchal or occipital).

These three large species can easily be distinguished from smaller species by the following highly visible characteristics: they all have only one postorbital spine (superciliary) and no spine is present on the occiput between the tympanum and nuchal crest. *A. murphyi* is similar to *A. nataliae*, differing from *A. capra* in that it has small, weakly keeled scales intermixed with large, keeled scales on the lateral and dorsal surfaces of the body.

The species combined into the *A. coronata* complex according to the results of the molecular genetic analysis (Figure 2A–C) differ sharply in body size: on the one hand, the large-sized agamid; and on the other hand, three small- and medium-sized species, two of which were recently described [11,30]. According to recent molecular data, *A. capra* is also most likely part of the *A. coronata* complex [30]. Morphologically, it is close to *A. murphyi*.

Detailed comparisons of *A. grismeri* with *A. coronata* and *A. cuongi* are presented in Table 4 [29]. It shows that *A. grismeri* differs from *A. coronata* Günther, 1861 by having a longer body (maximum SVL 113.09 mm versus 86.1 mm), a longer head, a larger orbit and tympanum, the presence of dorsal and nuchal crest spines, more subdigital lamellae on the fourth toe, fewer scales bordering the rostral scale, and fewer scales between the nasal and the rostral. It differs from *A. cuongi* Ngo, Le, Nguyen, Nguyen, Nguyen, Phan, Nguyen, Ziegler et Do, 2025 by having fewer scales bordering the rostral scale, lacking a diastema between nuchal and dorsal crests. Thus, this complex includes one of the largest species of the genus on the one hand, and the smallest representative of the genus *A. coronata* with a maximum length of 86.1 mm on the other.

**Figure 4:**
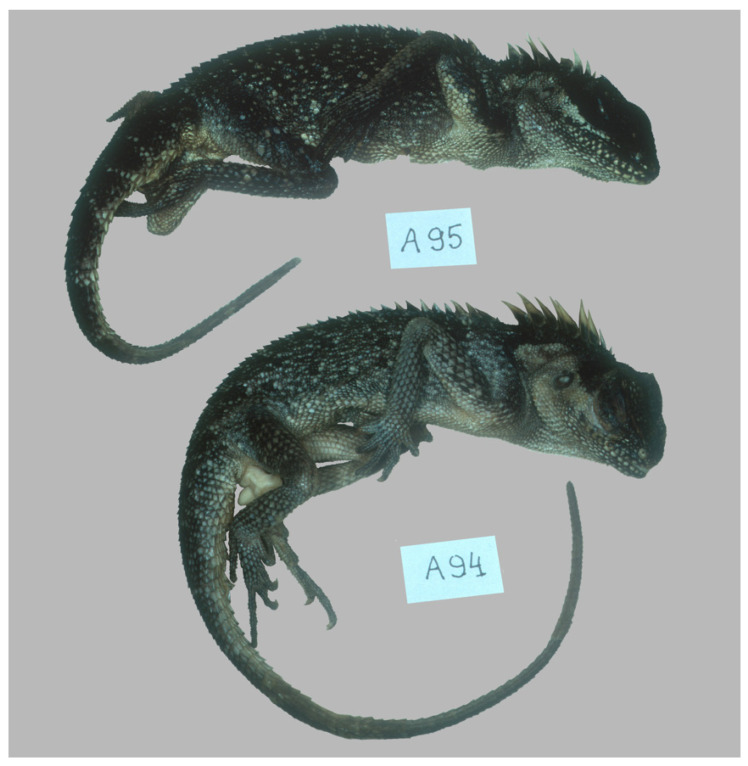
Specimens of *Acanthosaura murphyi*. SK-94 and SK-95 (HLMD-RA2969-26970) from Myanmar [14].

**Figure 5:**
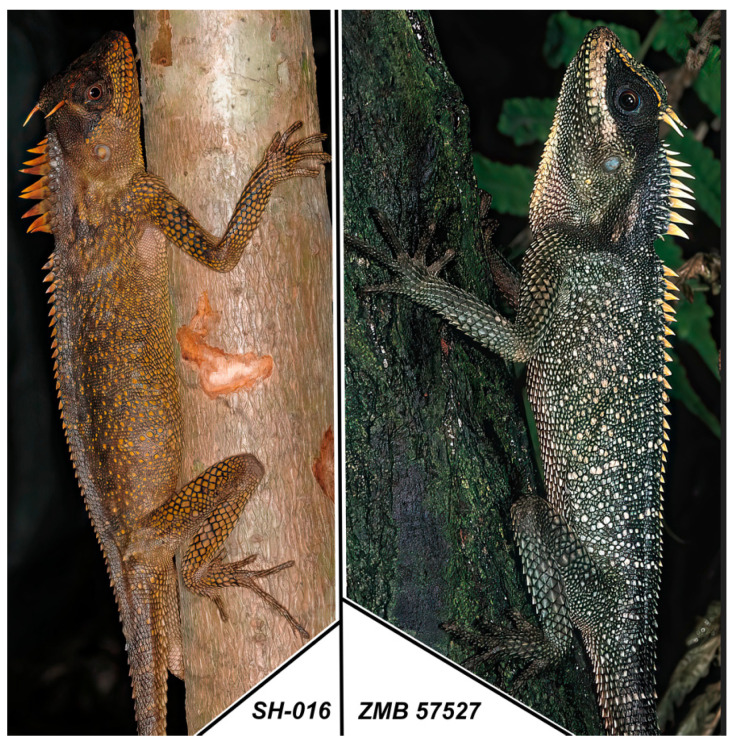
*A. murphyi* specimens in the wild: **left**, specimen with ILS H 2923 (voucher SH-016) from Song Hinh Commune, Phu Yen Province, Vietnam (2019); **right**, specimen with voucher ZMB 57527 from Vietnam (collected by U. Manthey in 1997, photo by U. Manthey).

**Figure 6:**
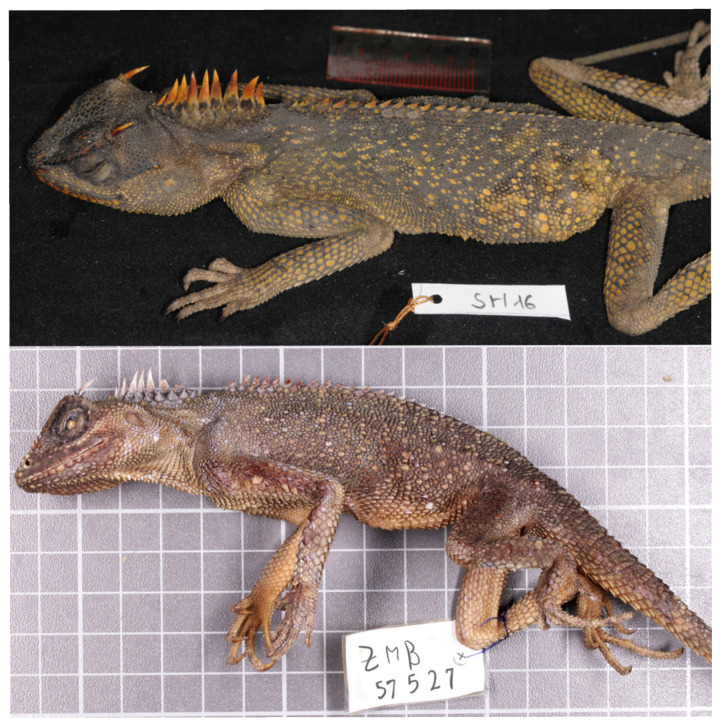
General view of *A. murphyi* specimens after preservation: **top**, specimen with ILS H 2923 (voucher SH-016) from Song Hinh Commune, Phu Yen Province, Vietnam (2019); **bottom**, specimen with voucher ZMB 57527 from Vietnam (collected by U. Manthey in 1997).

The same basic diagnostic features are evident in genotyped historical museum specimens (see Figure 4, Figure 5 and Figure 6, see Supplementary Table S2). Some of them have postorbital spines that appear as bumps, while others have no spines at all. These spines can easily break off in acanthosaurs, so this must be taken into account when collecting and keeping of lizards or preservation and storing of specimens.

## 4. Discussion

The genus *Acanthosaura* Gray, 1831 includes 22 species recently [1], divided into several species complexes. Two species, *A. aurantiacrista* Trivalairat, Kunya, Chanhome, Sumontha, Vasaruchapong, Chomngam et Chiangkul, 2020, and *A. cardamomensis* Wood, Grismer, Grismer, Neang, Chav et Holden, 2010, are represented only by ND2 sequences, while the *A. bintangensis* Wood, Grismer, Grismer, Ahmad, Onn et Bauer, 2009, *A. meridiona* Trivalairat, Sumontha, Kunya et Chiangkul, 2022, *A. phuketensis* Pauwels, Sumontha, Kunya, Nitikul, Samphanthamit, Wood et Grismer, 2015, and *A. titiwangsaensis* Wood, Grismer, Grismer, Ahmad, Onn et Bauer, 2009 lack any sequences in genetic databases, precluding definitive conclusions about their group assignments.

The other species of *Acanthosaura* are grouped into the following complexes according to molecular data [7]: (1) *armata*, which consists of *A. armata* (Gray, 1827), and *A. tongbiguanensis* Liu et Rao, 2019; (2) *capra*, which consists of *A. capra* Günther, 1861, and *A. nataliae* Orlov, Truong et Sang, 2006; (3) *crucigera*, which consists of *A. crucigera* Boulenger, 1885 and, possibly, *A. liui* Liu, Hou, Mo et Rao, 2020, (4) *lepidogaster*, which consists of *A. lepidogaster* (Cuvier, 1829), *A. brachypoda* Ananjeva, Orlov, Nguyen et Ryabov, 2011, and *A. longicaudata* Liu, Rao, Hou, Orlov, Ananjeva et Zhang, 2022; (5) *phongdienensis*, comprising *A. phongdienensis* Nguyen, Jin, Vo, Nguyen, Zhou, Che, Murphy et Zhang, 2019, and *A. rubrilabris* Liu, Rao, Hou, Orlov, Ananjeva et Zhang, 2022; (6) *prasina*, comprising a species *A. prasina* Ananjeva, Ermakov, Nguyen, Nguyen, Murphy, Lukonina et Orlov, 2020. The seventh, and most distant, compared to other acanthosaurs, group is the *A. coronata* complex, that includes (according to this study and our previous [8]) *A. coronata* Günther, 1861, *A. cuongi* Ngo, Le, Nguyen, Nguyen, Nguyen, Phan, Nguyen, Ziegler et Do, 2025, *A. grismeri* Le, Nguyen, Nguyen, Ziegler, Do et Ngo, 2025, and *A. murphyi* Nguyen, Do, Hoang, Nguyen, Mccormack, Nguyen, Orlov, Nguyen et Nguyen, 2018.

The molecular analysis showed that the topology of BI and ML phylogenetic trees constructed using three mitochondrial genes has a similar structure. The phylogeny based on the cytochrome *c* oxidase gene fragment (*COI*) yielded the best result for detection due to its highest representation in genetic databases. We supplemented the data for the rarest, yet most promising, *ND2* gene for our agama species, which may allow for a slightly more accurate identification of cryptic species in the future.

Analysis of the mitophylogenies confirms the species status of *A. murphyi*. This allows us to identify historical museum specimens from ZMB and HLMD that were previously misidentified or in doubt. Our analysis revealed that this group comprises specimens from Central Vietnam, which forms the basis for the species description of *A. murphyi*. The group also encompasses recent specimens of *A. murphyi* from Song Hinh Commune in Phu Yen Province, Vietnam, as well as museum specimens from Vietnam that were previously categorized as *A*. cf. *capra* [31] (fig. RA00028-4, p. 21). Additionally, the group includes *Acanthosaura* specimens from Myanmar, for which the precise locality is unknown (Figure 3). This has enabled us to corroborate our previous hypothesis [7] that *A. murphyi* belongs to the same species group as *A. coronata* and *A. cuongi*—the *A. coronata* complex.

An unexpected finding is the paraphyly of *A. capra*, which is represented by two groups (species) on the phylogenetic tree. In addition to *cyt b* sequences of specimens related to *A. nataliae* and sister to *A. lepidogaster* (AY572873-86), there is also an *A. capra* sequence (MK239022) within the studied complex that are sister to *A. murphyi*. This particular specimen formed the basis for considering *A. murphyi* as part of the *A. capra* group, a conclusion that was substantiated in the original species description [15]. It is known that *A. capra*, which is close to the *A. lepidogaster* complex, was collected in Krong Pa and Tram Lap, Gia Lai Province, Vietnam, whereas the agama from the *A. coronata* complex was caught in Bu Gia Map National Park, Binh Phuoc Province, Vietnam. Hopefully, further research and sequencing of material from the *terra typica* of *A. capra* will help determine which of these agamas belongs to the true *A. capra*.

## 5. Conclusions

This study provides a revision of the phylogenetic relationships within the *Acanthosaura coronata* complex, resolving long-standing ambiguities through an integrated molecular and morphological approach. Analysis of phylogeny based on three mitochondrial genes (*cyt b*, *COI*, and *ND2*) from both fresh field collections and historical museum specimens allows us to clarify the taxonomic status of several previously enigmatic lineages. Our phylogenetic analyses robustly confirm the distinct species status of *A. murphyi* and, for the first time, definitively place it within the *A. coronata* complex, alongside *A. coronata* and *A. cuongi*. This finding is consistently supported by tree topologies based on all three genetic markers and contradicts earlier morphological hypotheses that allied *A. murphyi* with *A. capra*.

The genetic data have enabled the taxonomic reassignment of historical museum specimens with uncertain or erroneous identifications. Specimens from the ZMB and HLMD collections, including those from Vietnam and an unknown locality in Myanmar, were unequivocally identified as *A. murphyi*. This expands the known distribution of *A. murphyi* considerably westward, suggesting it is not a narrow-range endemic but a more widely distributed species across central Vietnam and potentially into Myanmar. Our study also underscores the necessity of a multi-locus genetic framework for systematic revisions in *Acanthosaura*.

## Figures and Tables

**Table 1:** Primers used for the amplification and sequencing of mitochondrial DNA loci in the *Acanthosaura* species examined.

Locus	Primer Name	Sequence (5′—3′)	References
*COI*	VUTF	TGTAAAACGACGGCCAGTTCTCAACCAAYCAYAARGAYATYGG	[18]
	VUTR	CAGGAAACAGCTATGACTARACTTCTGGRTGKCCRAARAAYCA	
*cyt b*	L14841	CCATCCAACATCTCAGCATGATGAAA	[19]
	H151495	GCCCCTCAGAATGATATTTGTCCTCA	
*ND2*	METF6	AAGCTTTCGGGCCCATACC	[20,21]
	ALAr.2m	AAAGTGTCTGAGTTGCATTCRG	

## Data Availability

Sequences were deposited in GenBank NCBI (https://www.ncbi.nlm.nih.gov/) under the accession numbers PZ027938-46 (*COI*), PZ056157-72 (*ND2*), and PZ056173-77 (*cyt b*).

## Supplementary Materials

The following supporting information can be downloaded at https://www.mdpi.com/article/10.3390/ani16081261/s1, Table S1: Vouchers, NCBI GenBank accession numbers and localities of *Acanthosaura* specimens examined in this study; Table S2: Morphological comparison of specimens studied with *A. murphyi* and *A. coronata.*

## Abbreviations

The following abbreviations are used in this manuscript:

HLMD	Hessisches Landesmuseum Darmstadt, Germany
ILS H	Herpetological collection of the Institute of Life Sciences (ISL), Vietnam Academy of Science and Technology, Ho Chi Minh City, Vietnam
ZMB	Museum für Naturkunde Berlin Leibniz-Institut für Evolutions- und Biodiversitätsforschung, Invalidenstrasse 43, 10115 Berlin, Germany
ZMMU	Zoological Museum of Moscow State University, 125009 B. Nikitskaya 2, Moscow, Russia
