# Supplementary material for: Analysis of the Composition and Phylogenetic Relationships of the Acanthosaura coronata Complex Including Molecular Identification of Historical Specimens
Source: Animals (Basel). 2026 Apr 20;16(8):1261. doi: 10.3390/ani16081261 (PMC13113640; doi:10.3390/ani16081261)
Supplement: Supplementary file 1 [file animals-16-01261-s001.zip › Suppl. Table S1.pdf]

Suppl. Table S1. Vouchers, NCBI GenBank accession numbers and localities of *Acanthosaura* specimens examined in this study.

ID	Species	Vouchers	Country, Locality	COI	cyt b	ND2	Reference
1	*Acanthosaura coronata*	KH2	Viet Nam, Hon Ba Nature Reserve, Khanh Hoa prov.	**PZ027940**	-	-	This article
2	*Acanthosaura coronata*	R-11575.1	Viet Nam, Hon Ba Nature Reserve, Khanh Hoa prov.	**PZ027941**	-	-	This article
3	*Acanthosaura coronata*	R-11161.2	Viet Nam, Dong Nai prov., Nam Cat Tien National Park	**PZ027938**	-	-	This article
4	*Acanthosaura coronata*	R-11197.2	Viet Nam, Dong Nai prov., Nam Cat Tien National Park	**PZ027939**	-	-	This article
5	*Acanthosaura murphyi*	SH-016	Viet Nam, Tinh Phu Yen prov., Song Hinh distr.,Song Hinh Commune	**PZ027942**	**PZ056173**	**PZ056157**	This article
6	*Acanthosaura murphyi*	SH-017	Viet Nam, Tinh Phu Yen prov., Song Hinh distr.,Song Hinh Commune	**PZ027943**	**PZ056174**	**PZ056158**	This article
7	*Acanthosaura murphyi*	SH-018	Viet Nam, Tinh Phu Yen prov., Song Hinh distr.,Song Hinh Commune	**PZ027944**	**PZ056175**	**PZ056159**	This article
8	*Acanthosaura murphyi*	ZMB 57526	Viet Nam, south of the Mekong Delta	**PZ027945**	**PZ056176**	**-**	This article
9	*Acanthosaura murphyi*	ZMB 57527	Viet Nam, south of the Mekong Delta	**PZ027946**	**PZ056177**	**-**	This article
10	*Acanthosaura cuongi*	KKK53	Viet Nam, Gia La, K'Bang, Kon Ka Kinh National Park	PV646289	PV714672	**PZ056160**	Ananjeva et al., 2025; This article
11	*Acanthosaura cuongi*	KKK55	Viet Nam, Gia La, K'Bang, Kon Ka Kinh National Park	PV646288	PV658750	**PZ056163**	Ananjeva et al., 2025; This article
12	*Acanthosaura cuongi*	KKK108	Viet Nam, Gia La, K'Bang, Kon Ka Kinh National Park	PV646287	PV658753	**PZ056162**	Ananjeva et al., 2025; This article
13	*Acanthosaura cuongi*	KKK109	Viet Nam, Gia La, K'Bang, Kon Ka Kinh National Park	PV646287	PV658754	**PZ056161**	Ananjeva et al., 2025; This article
14	*Acanthosaura cuongi*	CMR88	Viet Nam, Kon Tum, Sa Thầy, Chu Mom Ray	PV646284	PV658746	**PZ056164**	Ananjeva et al., 2025; This article
15	*Acanthosaura cuongi*	CMR89	Viet Nam, Kon Tum, Sa Thầy, Chu Mom Ray	PV646285	PV658747	**PZ056165**	Ananjeva et al., 2025; This article
16	*Acanthosaura cuongi*	CMR90	Viet Nam, Kon Tum, Sa Thầy, Chu Mom Ray	PV646286	PV658748	**PZ056166**	Ananjeva et al., 2025; This article
17	*Acanthosaura coronata*	CTDN2	Viet Nam, Đồng Nai, Tân Phú, Cát Tiên National Park	PV646554	PV694983	**PZ056170**	Ananjeva et al., 2025; This article
18	*Acanthosaura coronata*	CTDN65	Viet Nam, Đồng Nai, Tân Phú, Cát Tiên National Park	PV646555	PV694984	**PZ056172**	Ananjeva et al., 2025; This article
19	*Acanthosaura coronata*	CTDN67	Viet Nam, Đồng Nai, Tân Phú, Cát Tiên National Park	PV646557	PV694985	**PZ056167**	Ananjeva et al., 2025; This article
20	*Acanthosaura coronata*	CTDN97	Viet Nam, Đồng Nai, Tân Phú, Cát Tiên National Park	PV646558	PV694986	**PZ056171**	Ananjeva et al., 2025; This article
21	*Acanthosaura coronata*	CTDN201	Viet Nam, Đồng Nai, Tân Phú, Cát Tiên National Park	PV646559	PV694987	**PZ056168**	Ananjeva et al., 2025; This article
22	*Acanthosaura coronata*	CTDN229	Viet Nam, Đồng Nai, Tân Phú, Cát Tiên National Park	PV646560	PV694988	**PZ056169**	Ananjeva et al., 2025; This article

Note. GenBank NCBI sequence numbers obtained in this study are highlighted in bold.
