# Supplementary material for: Analysis of the Composition and Phylogenetic Relationships of the Acanthosaura coronata Complex Including Molecular Identification of Historical Specimens
Source: Animals (Basel). 2026 Apr 20;16(8):1261. doi: 10.3390/ani16081261 (PMC13113640; doi:10.3390/ani16081261)
Supplement: Supplementary file 1 [file animals-16-01261-s001.zip › Suppl. Table S2.pdf]

Suppl. Table S2

Morphological comparison of specimens studied with *A. murphyi* and *A. coronata.*

Traits	*A. murphyi*		*A. murphyi* [15]				*A. coronata* [30]			
	ZMB 57527	ZMB 57526	Males (*n* = 7)		Females (*n* = 4)		Males (*n* = 39)		Females (*n* = 27)	
Morphometric traits										
SVL	130.3	126.7	81–127.3	108.4 ± 7.48	84.3–123	99.3 ± 8.33	55.6–79.9	65.9 ± 7.4	58.4–100.3	77.3 ± 11.7
TAL	172.2	179.2	121–195.8	164.4 ± 11.47	128.1–185	151.6 ± 12.09	67.1–119.9	89.1 ± 10.3	43.2–99.5	84.3 ± 11.5
TBW	14.4	13.6	10.3–18.3	14.6 ± 1.35	9–14.1	10.3 ± 1.27	5.48–11.33	8.3 ± 1.6	5.3–11.5	8.02 ± 1.73
TAL/SVL	1.32	1.41	1.48–1.54	1.5 ± 0.01	1.5–1.56	1.5 ± 0.01	–	–	–	–
HL	36.5	34.5	22.1–36.8	30 ± 2.13	24.1–34.1	28.5 ± 2.08	14–23.91	18.8 ± 2.4	15.1–27.42	21.5 ± 3.4
HW	19.2	18.9	14.8–24.6	19.9 ± 1.2	16.6–22.5	18.9 ± 1.31	11–17.25	13.4 ± 1.6	10.34–18.65	15.1 ± 2.2
HD	18.1	18.6	13.6–20.6	18.1 ± 1.03	15.2–20.1	17.2 ± 1.13	8.8–14.19	11.2 ± 1.4	8.99–15.19	12.4 ± 1.64
SL	14	13.2	8–15.3	11.2 ± 0.97	9.1–15.3	11.2 ± 1.45	5.2–9.94	7.25 ± 1.21	5.95–10.9	8.11 ± 1.15
ORBIT	11.8	11.1	6.4–12.2	10.1 ± 0.71	8.4–12.3	9.9 ± 0.89	3–8.19	5.67 ± 1.18	3.3–8.77	5.60 ± 1.41
EYE	8.2	8.6	4.8–9.1	6.8 ± 0.49	5.7–8.6	6.6 ± 0.68	–	–	–	–
TD	4.1	4.3	2.4–5.2	3.9 ± 0.38	2.2–5.1	3.8 ± 0.65	1.46–4.24	2.67 ± 0.56	1.8–4.06	3.04 ± 0.52
PS	7.58	NA	3.1–11.8	6.8 ± 1.04	3.5–5.6	4.5 ± 0.61	–	–	–	–
PS/HL	0.21	NA	0.1–0.34	0.2 ± 0.03	0.16–0.5	0.3 ± 0.11	–	–	–	–
NSL	7.04	8.3	3.9–14.9	9.2 ± 1.5	3.4–10.1	6 ± 1.44	–	–	–	–
NSL/HL	0.19	0.24	0.17–0.43	0.3 ± 0.03	0.14–0.3	0.2 ± 0.03	–	–	–	–
DS	4.1	3.8	2.1–10.5	4.5 ± 1.08	2.6–21	7.6 ± 4.48	–	–	–	–
WNC	3.3	3.9	1.8–4.8	3.4 ± 0.39	1.6–3	2.4 ± 0.33	–	–	–	–
WDC	3.7	3.4	2–3.3	2.6 ± 0.18	1.6–2.5	2.1 ± 0.2	–	–	–	–
DIAS	3.1	4.4	2–4.8	3.3 ± 0.4	2.2–4.2	3.2 ± 0.41	–	–	–	–
DIAS/HL	0.08	0.13	0.09–0.16	0.1 ± 0.01	0.08–0.13	0.1 ± 0.01	–	–	–	–
FOREL	47.3	44	31.3–56.6	48.9 ± 3.66	34.6–50.1	40.6 ± 3.47	27.68–41.4	35.5 ± 4.05	28.5–44.81	38.75 ± 4.8
HINDL	70.1	65	44.7–67.9	61 ± 3.49	25.6–68.4	47.7 ± 8.77	43.95–63.76	53.9 ± 5.8	42.58–67.96	56.2 ± 7.1
RW	5.1	4.1	2.1–4.3	3.4 ± 0.27	2.8–5.1	3.6 ± 0.52	–	–	–	–
RH	2.4	2	0.9–2	1.5 ± 0.14	1–2	1.4 ± 0.23	–	–	–	–
MW	2.3	1.6	1.1–1.8	1.6 ± 0.11	1.1–2.2	1.6 ± 0.25	–	–	–	–

MH	1.7	1.5	0.8–2	1.6 ± 0.14	1.4–1.8	1.6 ± 0.09	–	–	–	–
					Meristic traits (pholidosis)					
SN	10	10	8–9		8–8					
DIASN	7	6	4–8		5–6		6–11		7–10	
CS	12	12	12–14		11–12		8–13		7–11	
SUPRAL	12	13	12–14		12–13		10–13		10–14	
INFRAL	13	13	12–14		12–14		9–14		10–13	
VENT	59	57	54–65		55–61		46–63		40–57	
FI	19	18	15–18		15–18		11–17		12–16	
RS	8	7	7–9		7–8		2–6		3–5	
NS	8	8	6–9		7–9		5–9		7–10	
NCS	14	13	13–16		13–17		–		–	
NSCSL	7	7	7–10		7–9		–		–	
NR	3	3	3–4		3–4		–		–	
